# Harnessing Metabolic Insights: A Framework for Dietary Patterns in Chronic Disease Prevention and Management

**DOI:** 10.1016/j.advnut.2026.100607

**Published:** 2026-02-14

**Authors:** Fred K Tabung, Edward L Giovannucci

**Affiliations:** 1 Department of Internal Medicine, The Ohio State University College of Medicine, and Comprehensive Cancer Center, Columbus, OH, United States; 2 Department of Nutrition, Harvard T. H. Chan School of Public Health, Boston, MA, United States; 3 Department of Epidemiology, Harvard T. H. Chan School of Public Health, Boston, MA, United States

**Keywords:** metabolic dietary patterns, insulin resistance, chronic inflammation, insulin hypersecretion, major chronic disease, clinical translation

## Abstract

Metabolic dysfunction is a major driver of global chronic disease, yet current dietary guidance remains only loosely connected to the biological pathways that underlie these conditions. Historically, nutrition research emphasized individual nutrients or caloric content, overlooking the integrated metabolic effects of whole dietary patterns. Extensive research has linked dietary factors with chronic inflammation, insulin hypersecretion, and insulin resistance, with more recent studies synthesizing these associations into metabolically grounded dietary pattern indices, compared with the conventional nutrient- or calorie-focused approaches. Metabolic dietary patterns, empirically derived food-based indices that predict long-term metabolic biomarkers such as C-peptide and inflammatory cytokines, introduce mechanistic specificity into dietary assessment. This perspective reviews the development and evidence base of these patterns, compares them with conventional dietary pattern approaches, and synthesizes their nutritional characteristics and disease predictive capacity. Although many healthy dietary patterns are associated with improved chronic disease outcomes, metabolic dietary patterns show more consistent and robust associations, suggesting that targeting insulin resistance, a central hub connecting hyperinsulinemia, inflammation, and chronic disease, may better capture metabolically meaningful dietary variation. Because existing evidence is largely observational, we propose a structured translational framework for evaluating metabolic dietary patterns in clinical and community settings. Key tenets include preserving metabolic integrity; clarifying food and beverage intake targets; addressing items with uncertain or counterintuitive metabolic properties; accounting for food combinations and preparation methods; integrating food processing level; and ensuring cultural adaptability. This framework supports the translation of metabolic insights into actionable dietary guidance for precision prevention, clinical care, and public health.

**Statement of Significance**

This perspective provides the first integrative synthesis of metabolic dietary patterns—linking nutritional characteristics, biomarker specificity, mechanistic pathways, and disease prediction—and proposes a structured translational framework for adapting low-insulinemic and anti-inflammatory dietary patterns to clinical trials and public health practice.

## Introduction

During the second half of the 20th century, nutrient deficiency diseases declined sharply, aided by widespread consumption of low-cost fortified foods [1]. However, in many Western countries, this progress coincided with rising rates of obesity, type 2 diabetes, and cancer, alongside more heterogeneous trends in cardiovascular disease (CVD) [2]. Early research approached these chronic diseases through a reductionist focus on single nutrients—dietary fats, carbohydrates, and selected vitamins. Despite decades of large cohort studies and randomly assigned trials, this nutrient-centric paradigm provided limited evidence that any single macronutrient, particularly total fat or carbohydrate, explained substantial chronic disease risk [3], although some food-focused studies identified some protective (e.g., reduced intake of red and processed meats, increased intake of nuts) and hazardous (e.g., increased intake of baked goods rich in trans-fat) foods.

Nevertheless, the emphasis on minimizing dietary fat, especially saturated fat, strongly influenced public perception, the food industry, and federal guidelines, contributing to the proliferation of low-fat products and shaping the Dietary Guidelines for Americans, including the 2025–2030 Scientific Report [4], although the 2025–2030 guidelines diverged from the scientific report and recommended full-fat dairy [5]. Yet obesity and diabetes rates have continued to rise, and adherence to recommended dietary patterns remains modest [e.g., mean Healthy Eating Index-2020 (HEI-2020) ≈ 60/100 among United States adults] [6].

These trends, coupled with the underwhelming results of many nutrient-focused trials, led to a shift in the early 2000s toward evaluating overall dietary patterns rather than isolated nutrients [7]. Although an improvement, conventional patterning approaches have limitations. A priori patterns rely on foods historically linked to health; a posteriori patterns describe statistical eating clusters without biological rationale; and macronutrient-focused diets (low-fat, low-carbohydrate, ketogenic, etc.) often overlook noncaloric items such as coffee and lack mechanistic grounding [8].

Attempts to incorporate biology into dietary scoring have largely focused on acute postprandial responses, such as glycemic and insulinemic indices [[9], [10], [11], [12]]. Although useful, these indices capture short-term physiology rather than the chronic metabolic disturbances, persistent hyperinsulinemia, systemic inflammation, and insulin resistance, central to major chronic diseases. Animal models relying on simplified “high-fat” diets further limit translation because they do not reflect the complexity of human diets [13,14]. Despite broad agreement on the characteristics of healthy diets, evidence linking dietary patterns to major chronic diseases remains inconsistent, highlighting the challenge of identifying the dietary components most relevant to disease prevention and control.

Advances in dietary assessment, biomarker-rich cohort data, and biostatistical modeling now allow a more nuanced approach: dietary patterns defined by their long-term metabolic effects. These metabolic dietary patterns, derived from biomarkers of hyperinsulinemia, chronic inflammation, and related pathways, offer a biologically grounded framework for connecting habitual diet to disease pathophysiology.

In this perspective, we review and compare metabolic dietary pattern methodologies and synthesize their nutritional characteristics and disease predictive capacity relative to conventional approaches. We then outline a structured translational framework to support the application of metabolic dietary patterns in precision prevention, clinical counseling, and public health practice. The perspective offers the first integrative synthesis of metabolic dietary pattern indices, and the first structured translational framework for operationalizing them into clinical trials and guidelines.

## From Nutrients to Metabolic Pathways: Rationale for Metabolic Dietary Patterns

Although the dietary pattern approach aims to evaluate how overall diet influences health outcomes, conventional methods often fall short in predicting chronic disease. Most patterning strategies are designed either to reflect dietary guidelines or to describe prevailing eating behaviors, rather than to capture biologically meaningful variation linked to disease mechanisms. To address this gap, metabolic dietary patterns have emerged as a novel framework [[15], [16], [17]].

These patterns are built on the premise that dietary indices predictive of key metabolic pathways such as hyperinsulinemia, chronic inflammation, or dyslipidemia, may yield stronger disease prediction, particularly when these pathways are major determinants of risk. Examples include the empirical dietary index for hyperinsulinemia (EDIH) [16], the empirical dietary inflammatory pattern (EDIP) [17], the oxidative balance score [18], and others developed using similar principles. Rather than emphasizing macronutrient or micronutrient composition, or short-term postprandial responses, these indices reflect the long-term metabolic consequences of habitual diet.

Importantly, metabolic dietary patterns have demonstrated consistent associations with a wide range of chronic disease outcomes across multiple large prospective cohorts. In the sections that follow, we review the methodological approaches used to develop these patterns, with particular focus on those targeting insulin-related and inflammatory pathways, followed by a detailed comparison of the metabolic dietary indices themselves.

### Metabolic dietary patterns targeting insulin response

The glycemic index (GI) and glycemic load (GL) were the earliest indices used to estimate diet-driven insulin responses, though indirectly via postprandial glycemia [9,10]. To improve on these, the insulin index (II) and insulin load (IL) were developed as more direct measures of postprandial insulinemic responses [12]. However, all 4 indices reflect acute responses, which may be less relevant to the etiology of chronic disease. In contrast, the EDIH was created to estimate the long-term insulinemic potential of a habitual diet. Below, we review each approach and evaluate their utility for disease prediction.

#### Dietary GI/GL

Introduced in 1981, the GI classifies foods by their carbohydrate-induced effects on postprandial glucose [9]. The GL, introduced in 1997, incorporates both carbohydrate quality and quantity [10]. These indices validly capture postprandial glycemia but primarily reflect carbohydrate-driven insulin responses. Yet insulin secretion is influenced by additional macronutrients [19,20], micronutrients and bioactive compounds [21], and even noncaloric items such as coffee [22]—factors not accounted for by GI/GL.

Evidence from GI/GL interventions remains mixed. Some trials report improvements in fasting insulin, C-reactive protein (CRP), hemoglobin A1c, glucose, and total cholesterol [23], but these benefits often diminish in longer studies [24,25]. A meta-analysis of 29 randomized controlled trials found favorable effects on glycemic markers, lipids, body weight, and CRP only in trials under 12 wk [26]. Other analyses show no consistent effects on LDL cholesterol, HDL cholesterol, triglycerides, insulin, or adiposity [27], including among children with overweight or obesity [28]. Observational studies similarly report no consistent association between GI and BMI [27].

Reflecting these inconsistencies, the American Diabetes Association does not (yet) endorse a specific macronutrient distribution or GI/GL target due to variable definitions and limited clinical applicability [29]. In cancer research, GI/GL are not strongly linked to breast or colorectal cancer (CRC) incidence [30,31], though higher GI/GL have been associated with poorer prognosis and higher mortality among CRC survivors [32].

#### Dietary II/IL

Modeled after the GI, the dietary II, first introduced in 1997, directly assesses the postprandial insulinemic response to an isoenergetic portion of a reference food [11], and the IL, introduced in 2011, extends this concept to whole diets [12]. Unlike GI/GL, II and IL also capture insulin responses to low- or noncarbohydrate foods [12]. However, they remain short-term measures and do not account for insulin resistance, a key determinant of both postprandial and fasting insulin levels [33].

Most II/IL studies are cross-sectional, limiting causal inference. Nonetheless, findings consistently show that higher II and IL are associated with adverse metabolic markers, including hyperglycemia, larger waist circumference, and hypertriglyceridemia [34,35], elevated cholesterol, and higher blood pressure [36]. One prospective cohort reported an increased risk of type 2 diabetes with higher II/IL [37]. Although II/IL are not linked to incident cancer [38,39], several studies report associations with higher cancer-related and all-cause mortality [32,40,41].

#### Empirical dietary index for hyperinsulinemia

**FIGURE 1:**
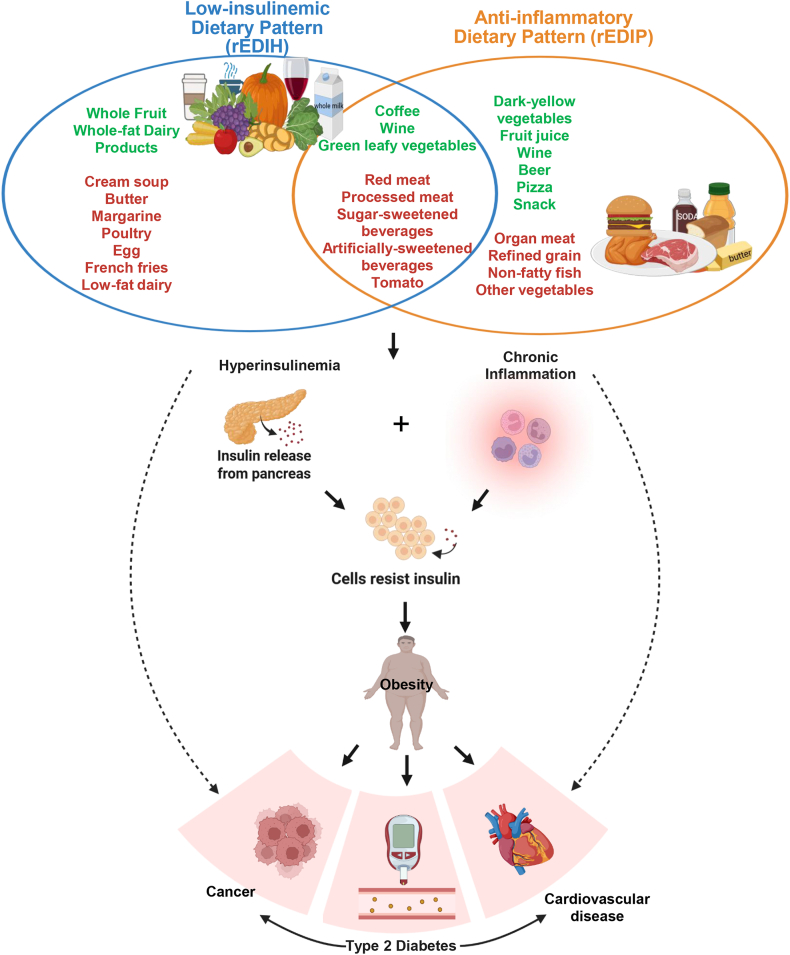
Core food and beverage components of the low-insulinemic (rEDIH) and anti-inflammatory (rEDIP) dietary patterns. This figure illustrates the overlapping yet distinct food components underlying each metabolic pathway. Colors indicate the direction of each food’s contribution to the respective index (red = higher insulinemic/inflammatory potential; green = lower potential). Components appearing in both indices are in the overlapping portion of the Venn diagram. rEDIH, reversed Empirical Dietary Index for Hyperinsulinemia; rEDIP, reversed Empirical Dietary Inflammatory Pattern.

Because chronic insulin exposure is more relevant to major chronic diseases than short-term postprandial responses, the EDIH was developed to capture long-term insulinemic potential using fasting plasma C-peptide, a robust marker of chronic insulin levels [16]. The index was derived using dietary data and fasting blood samples in the Nurses’ Health Study (NHS, *n* = 5812) and validated in NHS-II (*n* = 1002) and Health Professionals Follow-up Study (HPFS, *n* = 2632). The EDIH is a weighted sum of 18 food groups and beverages most predictive of fasting C-peptide, selected from 39 candidate food groups using regression modeling (Figure 1) [16]. EDIH reflects integrated insulin physiology, including secretion, resistance, and clearance [42,43], and has been extensively validated against multiple insulin-related biomarkers and major chronic disease endpoints [[44], [45], [46]].

EDIH, II/IL, and GI/GL measure distinct constructs of insulin-related dietary effects (Table 1). EDIH is essentially uncorrelated with II (*r* = –0.03) and IL (*r* = –0.02) [51,52], consistent with its focus on chronic insulin exposure. By contrast, IL correlates moderately to strongly with GI (*r* = 0.47) and GL (*r* = 0.78), reflecting shared postprandial glucose-insulin dynamics.

**TABLE 1:** Differences and similarities between the insulin-related dietary pattern indices

Characteristic	EDIH	II	IL	GI	GL
Biological construct assessed in the diet	Long-term insulin secretion and insulin resistance	Postprandial insulin secretion	Postprandial insulin secretion	Postprandial glucose secretion	Postprandial glucose secretion
Predicts fasting circulating C-peptide	Yes	No	No	No	No
Predicts non-fasting circulating C-peptide	Yes	No	No	Yes	Yes
Predicts (24-h) urinary C-peptide	Yes	Yes	Yes	No	Yes
Food predictors	Carbohydrate and noncarbohydrate foods	Carbohydrate and noncarbohydrate foods	Carbohydrate and noncarbohydrate foods	Carbohydrate containing foods only	Carbohydrate containing foods only
Must be a source of calories	No	Yes	Yes	Yes	Yes
Influences acute insulin secretion	Yes	Yes	Yes	Yes	Yes
Influences insulin resistance	Yes	No	No	No	No
Influences anthropometric parameters	Yes	No	No	No	No
Cardiometabolic disease risk prediction	Very robust	Weak	Weak	Weak	Weak
Mortality prediction	Very robust	Robust	Robust	Weak	Weak

Some of the data in this table were abstracted from references [[47], [48], [49], [50]].

Abbreviations: EDIH, empirical dietary index for hyperinsulinemia; GL, glycemic load; GI, glycemic index; II, insulin index; IL, insulin load.

**FIGURE 2:**
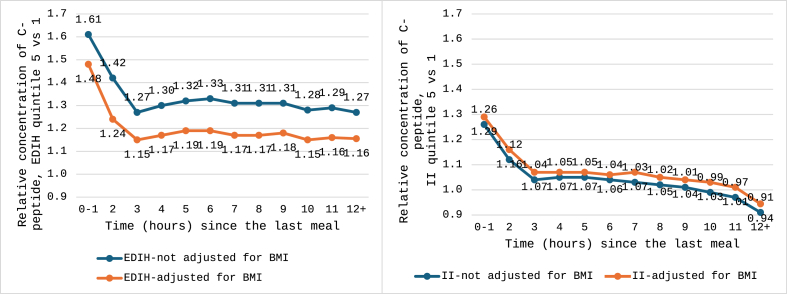
Relative concentrations of C-peptide over time since the last meal, in quintile 5 (most hyperinsulinemic) compared with quintile 1 (lowest insulinemia) of the EDIH and II. Data were abstracted from reference [43]. EDIH, Empirical Dietary Index for Hyperinsulinemia; II, insulin index.

Across all fasting and postprandial intervals (0–1 to ≥12 h since last meal), higher EDIH scores were associated with elevated C-peptide concentrations, with stronger associations among individuals with overweight or obesity [43]. These relationships remain significant after BMI adjustment, though attenuated. In contrast, dietary II shows weaker associations with C-peptide across meal-timing strata (Figure 2). Differences in C-peptide between extreme EDIH quintiles consistently exceed those for II. Supporting these findings, another study in males without diabetes found a significant positive association between EDIH and 24-h urinary C-peptide, a time-integrated index of insulin secretion [42].

Together, these findings indicate that EDIH captures a fundamentally different construct, chronic insulin exposure, whereas II, IL, and GI/GL primarily reflect short-term, meal-specific responses.

### Metabolic dietary patterns targeting chronic systemic inflammation

Several dietary indices have been developed to quantify the diet’s influence on chronic systemic inflammation, using different methodological approaches. Below, we compare 4 major indices, noting their shared themes and key distinctions.

#### Empirical dietary inflammatory pattern

Using a data-driven approach similar to EDIH, the EDIP was developed using dietary data and fasting plasma samples in the NHS (*n* = 5230) and validated in NHS-II (*n* = 1717) and HPFS (*n* = 4002), to capture associations between diet and chronic systemic inflammation. Reduced rank regression followed by stepwise linear regression was used to identify the dietary pattern most predictive of 3 fasting inflammatory markers: IL-6, CRP, and TNFα-R2 [53], and the resulting EDIP score is a weighted sum of 18 food groups and beverages, with weights derived from regression coefficients [53]. Higher EDIP scores reflect more proinflammatory diets. Several food groups associated with higher EDIP also appear in EDIH (Figure 1). The EDIP has been validated by its associations with inflammatory biomarkers and risk of inflammation-related chronic diseases in multiple cohorts [[44], [45], [46]].

#### Dietary inflammatory index

The dietary inflammatory index (DII) is a literature-derived, nutrient-based index [54], created from published associations between 45 nutrients, herbs/spices, and 6 inflammatory biomarkers (CRP, TNF-α, IL-6, IL-1β, IL-4, IL-10). Statistical transformations of these associations generated component-specific weights, which are applied to dietary intake data to compute the DII score; higher scores indicate more proinflammatory diets [54]. Methodologically, the DII differs substantially from the food-based EDIP [55]. Because 75% of its components are nutrients or bioactive substances, DII scores can be strongly influenced by nutrient supplements, complicating attempts to isolate dietary effects when total intake (foods + supplements) is used to compute the score. This may explain the modest correlations between DII and EDIP observed in 2 cohorts (*r* = 0.21 and 0.29) [55]. Use of total intake in most DII studies introduces additional complexity. When DII is computed using only food-derived intake, associations with health outcomes often weaken or become nonsignificant [[56], [57], [58], [59]]. Thus, supplements can influence DII both by altering scores directly and by adding behavioral confounding.

The DII’s nutrient-level structure offers cross-population comparability, as different foods can supply similar nutrients, but it overlooks the food matrix, which can markedly alter physiological effects. For example, yogurt and cheese share components with butter, yet fermentation, structure, and matrix differences lead to distinct metabolic associations [59,60]. Such interactions, often obscured in nutrient-based analyses, may contribute to the limited translational success of single-nutrient supplementation trials. In addition, it is easier to compute the DII in different populations as nutrients (compared with foods) are culturally neutral, for example, since its introduction in 2014, the DII has been applied in more than 1600 studies across numerous populations and health outcomes.

#### Dietary inflammation score

The dietary inflammation score (DIS) was derived using 19 foods, beverages, and 1 composite micronutrient supplement component, selected a priori based on biological plausibility and existing evidence [61]. Component weights were generated through linear regression relating each item to an inflammation biomarker score (CRP, IL-6, IL-8, IL-10). Higher DIS scores indicate more proinflammatory diets [61]. DIS and DII share several features: both rely on literature-based component selection and both include supplements. Correlations between DIS and DII were high across 3 cohorts (*r* = 0.59, 0.66, 0.66), whereas correlations with EDIP were more modest (*r* = 0.17, 0.23, 0.43) [61]. A key distinction is that DIS is primarily food-based, better preserving the context in which nutrients are consumed, whereas DII’s nutrient-driven structure ignores the food matrix. Supplement use influences DII scores substantially more than DIS, given that only one of DIS’s 19 components involves supplements [62,63]. To better isolate dietary effects, separating food-derived from supplement-derived intake scores remains advisable. DIS has shown associations with metabolic syndrome and type 2 diabetes in cross-sectional and case-control studies [64], and with CRC [63] and mortality [65] in prospective cohorts.

#### Anti-inflammatory diet index

The anti-inflammatory diet index (AIDI) was developed using dietary data and fasting blood samples from the Swedish Mammography Cohort (*n* = 3505) by identifying 20 foods that showed significant Spearman correlations with circulating CRP [66]. Higher AIDI scores reflect more anti-inflammatory diets, opposite in direction to DII, DIS, and EDIP. Unlike DII and DIS, AIDI does not include supplements; unlike DIS, its food components were empirically derived from observed biomarker associations rather than selected a priori. In derivation and food-based structure, AIDI most closely resembles EDIP, although the 2 indices have not yet been directly compared. A recent update of the AIDI in the Cohort of Swedish Men (*n* = 4432), using CRP and a broader array of inflammatory biomarkers and modified statistical methods, produced similar correlations with inflammatory markers, supporting the robustness of the construct [67]. AIDI has been applied mainly in CVD epidemiology, showing significant inverse associations with risks of abdominal aortic aneurysm, peripheral artery disease, heart failure, venous thrombosis, and all-cause mortality [[68], [69], [70], [71]].

In subsequent sections, we conduct a focused synthesis of EDIH and EDIP evidence because they are the empirically derived food-based dietary pattern indices that directly index 2 central metabolic pathways, chronic hyperinsulinemia and chronic systemic inflammation, and have accumulated the most extensive evidence base among such food-based indices in large prospective cohort studies, with biomarker and major chronic disease outcomes. Although other indices, such as the DII, have also been widely studied, these are predominantly nutrient-based, whereas EDIH and EDIP uniquely integrate whole-food patterns with metabolic biomarkers, making them relatively easier to translate to dietary pattern interventions. For clarity, we use reverse empirical dietary index for hyperinsulinemia (rEDIH) and reversed empirical dietary inflammatory pattern (rEDIP) to indicate reverse-scored versions of the EDIH and EDIP. Scores are reversed so that higher values reflect lower insulinemic or lower inflammatory potential, respectively, facilitating direct comparison with conventional dietary indices in which higher scores represent healthier diets.

## Synthesis of the Nutritional Characteristics of Metabolic (Insulinemic and Inflammatory) Dietary Patterns in Relation to Conventional Dietary Patterns

Conventional dietary patterns derive their food and nutrient composition a priori, grounded in prevailing nutrition science and assumptions about how specific nutrients influence disease risk, for example, saturated fat’s effect on LDL cholesterol or sodium and potassium’s relation to blood pressure. In contrast, the rEDIH and rEDIP were developed using a nutrient-independent, data-driven approach, identifying foods solely based on their empirical associations with metabolic biomarkers. Consequently, the food combinations that define these metabolic patterns do not always mirror conventional dietary guidelines.

**FIGURE 3:**
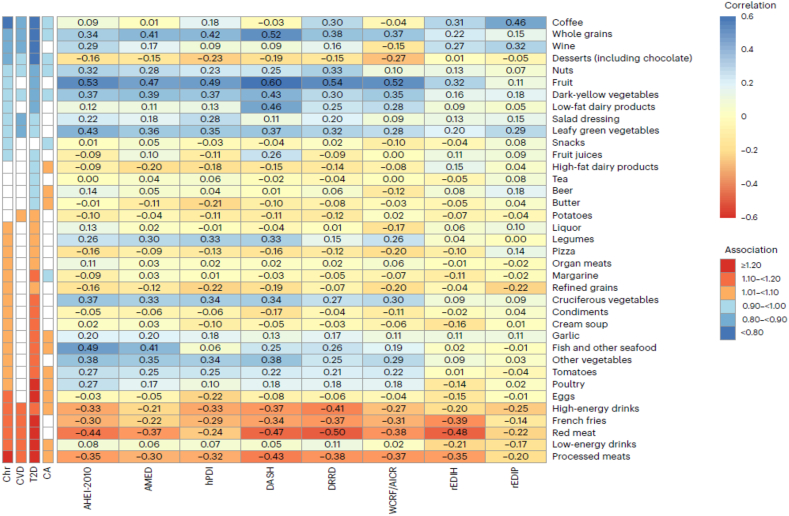
Spearman’s correlations between energy-adjusted cumulative average dietary patterns index scores and food groups are shown, with correlation coefficients highlighted in color. Food groups are ordered based on the HRs for their associations with major chronic disease outcomes: major Chr, major CVD, T2D, and total CA, comparing the 90th with the 10th percentile. The figure conveys 2 related messages: (A) HRs indicate how individual foods are associated with disease risk across dietary patterns, allowing comparison of disease associations between patterns; (B) correlations show how foods relate to different dietary pattern scores, facilitating comparison of food contributions across patterns. Significant HRs (*P* < 0.05) are highlighted in color according to magnitude. (Figure adapted from Wang et al. [46].) AHEI-2010, Alternative Healthy Eating Index-2010; AICR, American Institute for Cancer Research; AMED, alternative Mediterranean dietary pattern; CA, cancer; Chr, chronic; CVD, cardiovascular disease; DASH, dietary approaches to stop hypertension; DRRD, diabetes risk reduction diet score; hPDI, healthy plant-based dietary index; HRs, hazard ratios; rEDIH, reverse Empirical Dietary Index For Hyperinsulinemia; rEDIP, reversed Empirical Dietary Inflammatory Pattern; T2D, type 2 diabetes; WCRF, World Cancer Research Fund.

As shown in Table 2 and Figure 3, several food groups consistently appear, positively or negatively, across both metabolic dietary patterns (rEDIH, rEDIP) and conventional patterns including the HEI, Alternative HEI (AHEI), dietary approaches to stop hypertension (DASH) diet, Alternative Mediterranean Diet (AMED), healthy plant-based index (hPDI), diabetes risk-reduction diet (DRRD), and WCRF/AICR guidelines. Foods for reduction across patterns include red and processed meats, sugar-sweetened beverages, eggs, and French fries. Conversely, leafy green vegetables, dark-yellow vegetables, fruits, nuts, and whole grains are widely encouraged. Some distinctions remain: cruciferous vegetables feature prominently in conventional patterns but contribute less to metabolic dietary patterns compared with leafy greens and dark-yellow vegetables. Dairy intake highlights another divergence. Whereas conventional patterns typically emphasize low-fat or nonfat dairy, metabolic dietary patterns favor whole-fat dairy and restrict low- or nonfat varieties. Similarly, foods such as poultry, tomatoes, and nonfatty fish, commonly promoted in conventional guidelines, are limited in rEDIH and rEDIP. Metabolic patterns also incorporate noncaloric items often overlooked in conventional models: artificially sweetened beverages (ASBs) are discouraged, whereas coffee is emphasized.

**TABLE 2:** Distribution of mean food intakes, macronutrients and micronutrients in the highest (low insulinemic, low inflammatory) and lowest (high insulinemic, high inflammatory) quintiles of rEDIH and rEDIP dietary patterns, compared with overall dietary quality (HEI-2015) among 1 million males and females in North America and Europe (data abstracted from Shi et al. [45])

Dietary variable	rEDIH			rEDIP			HEI-2020		
	High insulinemic	Low insulinemic	Difference (low–high)	High inflammatory	Low inflammatory	Difference (low–high)	Low overall dietary quality	High overall dietary quality	Difference (high–low)
Dietary score median	–1.18	1.15	2.33	–1.14	1.09	2.23	51.9	76.7	24.8
Food intake, servings/wk									
Green leafy vegetables	5.98	9.79	3.80	5.66	10.87	5.21	4.63	9.32	4.69
Dark yellow vegetables	2.73	3.40	0.67	2.40	3.76	1.36	1.64	4.06	2.42
Other vegetables	6.19	7.10	0.92	6.05	6.47	0.42	4.22	7.38	3.16
Tomato	4.26	4.51	0.25	4.53	4.23	–0.30	3.17	4.80	1.63
Whole fruit	8.83	15.61	6.78	10.7	13.0	2.36	6.49	16.3	9.80
Fruit juice	6.25	9.47	3.22	6.11	9.34	3.23	4.59	9.98	5.39
Full fat dairy	10.2	14.5	4.31	10.7	13.0	2.28	12.4	9.91	–2.52
Pizza	0.72	0.68	–0.03	0.61	0.91	0.30	0.76	0.47	–0.29
Wine	1.39	4.25	2.86	1.20	4.90	3.70	1.71	2.67	0.97
Coffee	17.5	19.8	2.25	12.7	25.7	13.0	19.5	16.3	–3.13
Tea	6.90	7.92	1.02	5.60	8.99	3.38	6.81	7.88	1.08
Beer	2.21	3.77	1.56	1.15	5.44	4.29	1.91	2.06	0.15
Total alcohol	8.12	11.0	2.91	6.43	13.5	7.08	6.52	8.99	2.48
Snacks	3.36	3.19	–0.17	2.98	3.49	0.51	2.93	3.13	0.19
Processed meat	12.6	4.48	–8.09	11.8	5.21	–6.58	9.85	5.07	–4.78
Read meat	33.2	10.0	–23.2	28.1	12.9	–15.2	21.5	13.0	–8.51
Organ meat	1.01	0.53	–0.48	0.90	0.55	–0.35	0.80	0.47	–0.33
Poultry	18.6	7.83	–10.8	13.4	11.7	–1.65	9.24	13.4	4.15
Egg	3.01	2.05	–0.96	2.73	2.27	–0.46	2.60	1.91	–0.69
White fish	8.84	3.62	–5.22	10.25	3.26	–6.99	3.74	7.02	3.28
Diet soda	1.86	0.70	–1.16	2.19	0.59	–1.60	0.95	1.08	0.13
Regular soda	21.1	8.28	–12.8	26.6	6.50	–20.1	14.8	9.60	–5.24
Margarine	20.1	10.5	–9.55	13.9	14.5	0.64	11.4	16.0	4.62
Butter	10.15	6.20	–3.94	6.67	8.81	2.14	12.19	3.58	–8.61
French fries	1.53	0.48	–1.05	1.07	0.74	–0.34	0.95	0.65	–0.30
Low fat dairy	2.59	4.29	1.70	2.42	4.33	1.92	1.79	5.50	3.71
Refine grain	28.0	31.9	3.89	37.3	22.8	–14.5	35.0	20.8	–14.2
Cream soup	0.38	0.22	–0.16	0.29	0.27	–0.02	0.26	0.24	–0.02
Nutrients									0.00
Total carbohydrate (g)	114	133	19.3	122	125	2.93	119	135	15.7
Cholesterol (mg)	153	93.4	–59.7	140	105	–35.0	143	100	–43.2
Total saturated fats (g)	13.0	11.0	–2.03	12.1	11.4	–0.74	14.7	9.36	–5.30
Total fat (g)	39.3	32.5	–6.81	37.4	33.6	–3.80	39.7	31.7	–7.92
Total transfat (g)^f^	1.97	1.40	–0.57	1.72	1.49	–0.23	1.82	1.46	–0.36
Total protein (g)	42.9	34.2	–8.73	41.3	35.3	–5.99	36.8	39.5	2.61
Total dietary fiber (g)	9.45	12.11	2.66	9.82	11.63	1.81	8.22	13.63	5.41
Calcium (mg)	389	513	124	416	482	65.1	430	502	71.9
Magnesium (mg)	160	187	27.3	157	191	33.6	148	204	55.5
Sodium (mg)	1520	1281	–239	1450	1328	–122	1467	1322	–145
Vitamin A, RAE (μg)	2575	3501	926	2343	3933	1590	2018	4006	1988
Vitamin B-12 (μg)	3.43	2.37	–1.06	3.38	2.41	–0.97	2.81	2.94	0.14

Data are abstracted from Shi et al. [45], pooling from 6 cohorts that included 1 million adults. The 6 cohorts are NIH-AARP Diet and Health Study (AARP), ATBC, EPIC, MESA, PLCO cancer cohort, and SCCS.

Abbreviations: AARP, American Association of Retired Persons; ATBC, Alpha-Tocopherol, Beta Carotene Cancer Prevention Study; EPIC, European Prospective Investigation into Cancer and Nutrition; HEI-2015, Healthy Eating Index-2015; MESA, multiethnic study of atherosclerosis; PLCO, prostate, lung, colorectal, and ovarian; rEDIH, reverse Empirical Dietary Index for Hyperinsulinemia; rEDIP, reversed Empirical Dietary Inflammatory Pattern; SCCS, Southern Community Cohort Study.

When associations with metabolic disease risk are examined across food groups (left panel of Figure 3), empirical patterns tend to align more closely with chronic disease outcomes than with conventional dietary patterns. Metabolic dietary patterns themselves show broad similarity, with moderate correlations (Spearman *r* = 0.50–0.70) in previous studies [45]. Nine food groups are consistently observed: 5 associated with higher risk (red meat, processed meat, nonfatty fish, sugar-sweetened beverages, ASBs) and 4 associated with lower risk (wine, coffee, leafy green vegetables, whole-fat dairy). Each index retains unique contributors; for example, eggs, poultry, cream soups, butter, and margarine are more strongly related to rEDIH than to rEDIP.

An important consideration not explicitly incorporated into the development of rEDIH and rEDIP is food processing. Many foods identified as harmful in these patterns—French fries, SSBs, ASBs, are also classified as ultraprocessed foods (UPFs) under NOVA [72]. Although processing level was not a criterion in derivation, the overlap suggests that part of the adverse effects of UPFs may be mediated through chronic inflammation and hyperinsulinemia. Emerging evidence indicates that UPFs exert metabolic effects beyond nutrient composition, potentially through additives, altered food matrices, or impacts on the gut microbiome [73,74]. Still, UPFs vary widely in health effects, underscoring the need for more nuanced classification within dietary pattern indices.

Table 2 also summarizes the nutrient profiles of low-insulinemic or anti-inflammatory dietary patterns compared with hyperinsulinemic or proinflammatory patterns [45]. Although certain foods contributing to lower risk (e.g., whole-fat dairy, some mixed dishes such as pizza) are relatively high in fat, the overall macronutrient profile of diets with more favorable rEDIH/rEDIP scores reflects food-level substitutions rather than extreme macronutrient shifts. Specifically, these diets tend to feature moderately lower total and saturated fat from animal sources, lower dietary cholesterol, moderately lower animal protein, moderately higher total carbohydrate, and higher dietary fiber. This pattern arises, for example, from greater intake of whole-fat dairy combined with lower consumption of red and processed meats.

Regarding micronutrients, low-insulinemic or anti-inflammatory diets are associated with higher intake of several underconsumed nutrients in the United States diet [75] (Table 2). Notably, these favorable micronutrient patterns have been consistently observed across diverse study populations [45]. Thus, although rEDIH and rEDIP were developed through a food-based, nutrient-agnostic process, the dietary patterns most predictive of chronic hyperinsulinemia or systemic inflammation inherently reflect high-quality overall nutrition.

### Comparison of the disease predictive ability of the metabolic dietary patterns with the conventional dietary patterns

A large comparative study [46] used a standardized prospective approach to evaluate the disease-predictive ability of low-insulinemic (rEDIH) and anti-inflammatory (rEDIP) patterns alongside 6 conventional dietary patterns (AHEI-2010, AMED, hPDI, DASH, DRRD, WCRF/AICR). The composite outcome included incident type 2 diabetes, CVD, and cancer among 205,852 adults followed for up to 32 y. Although greater adherence to all 6 conventional patterns was associated with lower risk of major chronic disease, the largest risk reductions occurred for rEDIH and rEDIP, both for the composite outcome and for each disease individually (Table 3). These findings suggest that whereas conventional patterns are beneficial, dietary patterns specifically calibrated to insulinemic and inflammatory pathways may offer superior predictive power and may inform future prevention-oriented dietary guidelines.

**TABLE 3:** A comparison of the disease preventive ability of the conventional dietary patterns compared with the metabolic dietary patterns

Dietary pattern	Major chronicdisease risk (%)	Type 2 diabetesrisk (%)	Cardiovasculardisease (%)	Coronary arterydisease (%)	Stroke (%)	Obesity-relatedcancers (%)
rEDIH	–32	–65	–32	–37	–24	–7
rEDIP	–30	–62	–31	–35	–23	–8
DRRD	–23	–44	–27	–29	–24	–7
AHEI–2010	–17	–38	–23	–28	–16	–2^∗^
AMED	–16	–29	–20	–22	–16	–3^∗^
DASH	–17	–34	–19	–21	–16	–4^∗^
hPDI	–17	–30	–16	–20	–9	–6

Values are multivariable-adjusted (MV)-adjusted hazard ratios, meta-analyzed across 3 large cohorts including 205,852 males and females, and comparing the 90th compared with 10th percentile of dietary pattern index scores. All values are statistically significant (*P* > 0.05) except ^∗^. Major chronic disease was defined to include incident major cardiovascular disease, type 2 diabetes, total cancer. low insulinemic dietary pattern or rEDIH; anti-inflammatory dietary pattern or rEDIP score; DRRD score, included higher intakes of cereal fiber, nuts, coffee, and PUFA:SAFA ratio; lower intakes of trans-fat, SSB, red and processed meats; AHEI-2010, alternative healthy eating index-2010; AMED score; DASH diet; hPDI, included higher intakes of whole grains, fruits, vegetables, nuts, legumes, vegetable oils, tea/coffee; and lower intakes of fruit juices, SSBs, refined grains, potatoes, sweets), butter/lard, dairy, egg, fish/seafood, and meats. Data were abstracted from the MV model in Table 2 of Wang et al., NatMed [46].

Abbreviations: AHEI-2010, Alternative Healthy Eating Index-2010; AMED, alternative Mediterranean dietary pattern; DASH, dietary approaches to stop hypertension; DRRD, diabetes risk reduction diet score; hPDI, healthy plant-based dietary index; MV, multivariable; SAFA, safety assessment of foreign aircraft; SSB, sugar-sweetened beverages.

#### Body weight

Prospective analyses across six 4-y intervals (24 y total) show that long-term changes in EDIH and EDIP scores are associated with distinct weight trajectories. Shifts toward more low-insulinemic and less inflammatory diets were linked to significantly less weight gain over time [76]. Comparable analyses of AHEI, AMED, and DASH yielded similar, though slightly smaller, associations: each 1-SD increase in AHEI score corresponded to 0.47 kg less weight gain per 4-y period [77], with similar findings for DASH and modestly weaker associations for AMED [77].

Although the absolute weight differences appear small, they accumulate meaningfully. In Ref. [76], females shifting toward the most hyperinsulinemic diet gained 1.50 kg per 4-y interval, compared with 0.75 kg among those adopting the lowest-insulinemic diet, a potential halving of weight gain over 24 y (from 9.0 to 4.5 kg). Thus, while higher overall dietary quality reduces weight gain, specifically lowering dietary insulinemic and inflammatory potential may confer additional benefits.

Although dietary pattern research typically adjusts for total energy intake to isolate dietary composition (implying calorie neutrality), this may not reflect real-world effects. Certain patterns may influence appetite, satiety, or energy density. Notably, rEDIH and rEDIP show stronger associations with BMI and weight change than many conventional patterns [76,77], suggesting that diets with low insulinemic or anti-inflammatory potential may support energy balance by improving satiety or reducing energy density, in addition to modulating metabolic pathways.

#### Type 2 diabetes

Dietary patterns strongly influence type 2 diabetes risk [[78], [79], [80], [81]]. Across multiple prospective cohorts, higher adherence to rEDIP and rEDIH has been associated with 29%–51% and 30%–48% lower risk of type 2 diabetes, respectively [43,44,78,79], substantially greater reductions than those from conventional dietary patterns (Table 3). Metabolomic analyses provide mechanistic insight: 27 plasma metabolites were linked to both rEDIP and inflammatory biomarkers, and 21 metabolites to rEDIH and C-peptide. Composite metabolomic scores reflecting lower inflammation and reduced insulin secretion were associated with >3-fold lower diabetes risk [82], suggesting that rEDIP and rEDIH influence disease risk partly through favorable shifts in the metabolome.

#### Cardiovascular disease

In the comparative study cited in Ref. [46], higher adherence to rEDIH and rEDIP was associated with 32% and 31% lower risk of overall CVD, respectively, compared with more modest reductions from conventional dietary patterns (16% for hPDI to 27% for DRRD). All estimates were statistically significant [46]. Furthermore, validation in a biomarker subsample of 33,719 participants showed that higher rEDIP was associated with lower proinflammatory markers, increased adiponectin, and a more favorable lipid profile (*P* < 0.001) [83], which in turn predicted lower risk of CVD, CHD, and stroke [83]. Higher rEDIH was additionally associated with lower all-cause, CVD, and cancer mortality [84].

#### Digestive system conditions including cancer and metabolically driven liver disease

Both rEDIH and rEDIP have been associated with a lower risk of several gastrointestinal conditions, including diverticulitis [85,86], CRC [[87], [88], [89], [90], [91], [92]], and other digestive cancers [93,94], as well as metabolically driven liver diseases such as nonalcoholic fatty liver disease (NAFLD; now termed metabolic dysfunction-associated steatotic liver disease) [95], and hepatocellular carcinoma [96]. Higher rEDIH and rEDIP scores were associated with an 18%–24% lower risk of diverticulitis in both sexes, partly mediated by metabolomic signatures. Consistent with its insulin resistance-driven etiology, rEDIP was also linked to lower incident NAFLD and reduced progression to cirrhosis [95].

For CRC, rEDIP was associated with a 24% lower overall risk—18% among females and 31% among males [88]. rEDIH also showed protective associations, particularly among females. Sex-specific differences in diet-CRC associations are well documented, with stronger estimates typically observed in males [87]. Recent evidence from the large multicohort data harmonization study further strengthens these findings. In an analysis of >1 million adults across 6 international cohorts, both the rEDIH and rEDIP dietary patterns retained robust associations with CRC risk despite substantial heterogeneity in dietary assessment methods and food environments [45]. This cross-cohort consistency highlights the biological relevance of metabolic pathway-based dietary scoring across both gastrointestinal and metabolically driven liver outcomes.

Inflammation-related molecular subtypes further refine risk patterns. Anti-inflammatory diets (higher rEDIP scores) were associated with a 39% lower risk of *Fusobacterium nucleatum*–positive proximal colon tumors, but not *F. nucleatum*–negative tumors [89]. rEDIP was also associated with lower incidence [90] and mortality [91] from CRC characterized by low lymphocytic infiltration; tumors are considered more aggressive. These findings support the hypothesis that habitual anti-inflammatory diets may enhance antitumor immunity and influence microbial dynamics, thereby reducing the risk of aggressive CRC.

## Framework for Translating Metabolic Dietary Patterns into Clinical Intervention Trials, Dietary Counseling and Public Health Promotion

Robust epidemiologic evidence links low-insulinemic (rEDIH) and anti-inflammatory (rEDIP) dietary patterns to lower risk of multiple chronic diseases, positioning these empirically derived patterns as promising candidates for pathway-targeted dietary interventions. Realizing this potential requires moving beyond their original derivation in United States cohorts and explicitly addressing issues of generalizability, cultural relevance, and implementation feasibility. Although developed in North American populations, the pathways they capture, chronic hyperinsulinemia and systemic inflammation, are universal. Findings from harmonized analyses of >1 million adults across diverse food environments demonstrate that insulinemic and inflammatory dietary patterns retain strong associations with CRC risk even when applied across heterogeneous dietary assessment tools [45]. This underscores the cross-population robustness of the underlying metabolic pathways while highlighting the need for recalibration, not direct transplantation, across cultures.

Translating empirically derived patterns into actionable recommendations raises questions about dietary granularity. The 18 foods and beverages that compose rEDIH and rEDIP are representative predictors, not prescriptive lists. Effective translation requires expanding these components to culturally relevant equivalents with similar metabolic properties, even when preparation methods, consumption patterns, or agricultural conditions vary. This maintains biological fidelity while improving real-world feasibility.

**FIGURE 4:**
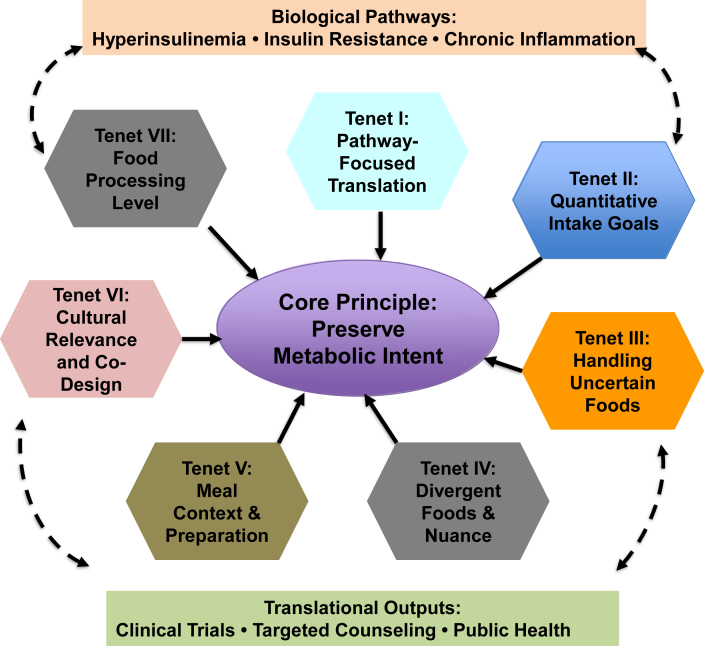
Translational framework for adapting metabolic dietary patterns (rEDIH, rEDIP) into clinical and public health interventions. The figure illustrates 7 key tenets guiding the translation of metabolic dietary patterns from epidemiologic discovery into practical implementation. The framework begins with pathway-based foundations (hyperinsulinemia, insulin resistance, chronic inflammation), incorporates quantitative targets, addresses foods with uncertain or mixed metabolic properties, integrates meal context and cultural heterogeneity, incorporates food processing level, and culminates in community-engaged adaptation. These tenets collectively support the operationalization of metabolic dietary patterns for clinical trials, dietary counseling, and public health strategies. rEDIH, reversed Empirical Dietary Index for Hyperinsulinemia; rEDIP, reversed Empirical Dietary Inflammatory Pattern.

This framework outlines 7 principles for operationalizing metabolic dietary patterns in research, clinical counseling, and public health. It is particularly relevant for subgroups seeking targeted dietary strategies, such as: *1*) individuals aiming to maintain weight loss after pharmacologic discontinuation, *2*) adults with diabetes or prediabetes seeking sustainable nonpharmacologic approaches to glycemic control, *3*) people undergoing cancer treatment desiring adjunctive dietary strategies, etc. Each principle is summarized below and illustrated in Figure 4.

### Tenet I: anchor dietary pattern translation in underlying biological pathways rather than on specific foods

Translation must prioritize the metabolic pathways targeted—chronic hyperinsulinemia and systemic inflammation—rather than replicate the exact foods identified in United States cohorts. The predictive biomarkers underlying rEDIH and rEDIP transcend food cultures, and the harmonized analyses across international cohorts confirm that these pathway-based patterns remain predictive across widely differing dietary landscapes [45]. The goal is to identify culturally relevant foods that exert similar metabolic effects, rather than attempting an exact replication of the original 18 components. A pathway-first approach preserves mechanistic integrity and facilitates culturally sensitive adaptation. In practice, this initial mapping involves grouping foods by processing level, preparation method, and typical consumption context, and assigning them to dominant hypothesized metabolic pathways rather than fixed nutrient thresholds, allowing adaptation across dietary assessment instruments and populations.

### Tenet II: define quantitative intake goals that are flexible, culturally adaptable, and metabolically grounded

Consistent application of metabolic dietary patterns requires approximate quantitative targets for key food groups. Standard portion sizes anchored to a reference energy intake (e.g., 2000 kcal/d) provide structure for guidance, intervention fidelity, and food labeling, while allowing individuals to adjust for their own energy needs. Although foods within a given group are not metabolically identical, grouping reflects directionally consistent effects on insulinemic and inflammatory pathways, with differences primarily in magnitude rather than direction. At the population level, such grouping improves the stability and generalizability of dietary pattern scores [45]. Quantitative targets should be mapped onto culturally relevant foods with analogous metabolic effects and interpreted as flexible reference ranges rather than fixed prescriptions. Insulinemic and inflammatory dietary patterns retain predictive value across populations with nonidentical dietary data [45], supporting adaptation across dietary cultures and individual physiological needs. Targets may be further refined for subgroups such as older adults, individuals with diabetes, or those in weight-maintenance phases.

### Tenet III: establish a systematic evidence-based approach for foods with uncertain or mixed metabolic properties

Many foods—composite dishes, culturally specific items, novel products—lack direct evidence of their insulinemic or inflammatory potential. A systematic approach can expand the patterns while protecting biological integrity: *1*) *Draw from mechanistic and category-level evidence* [59,60,97] when direct data are limited. *2*) *Leverage granular dietary data linked to biomarkers* (e.g., 24-h recalls) to classify less-studied foods. Cross-cohort analyses [45] show that metabolic directionality can be inferred even when food-level detail varies. *3*) *Create a temporary “uncertain” category* for items lacking evidence, updating classification as new biomarker or pilot data emerge. This approach preserves mechanistic fidelity while allowing for culturally broader applications.

### Tenet IV: translate foods that diverge from conventional guidance, with nuance, mechanistic context, and personalization

Some foods central to rEDIH and rEDIP diverge from prevailing dietary guidelines, requiring nuanced translation: *1*) *Whole-fat dairy—*although often discouraged in standard guidelines (until the 2025–2030 Dietary Guidelines for Americans), whole-fat yogurt and cheese score favorably, likely due to fermentation, matrix effects, and dairy-specific bioactives. *2*) *Moderate wine consumption:* wine scores favorably but conflicts with cancer-prevention guidelines. Translation should incorporate cultural norms, genetic differences in alcohol metabolism, and provide nonalcohol alternatives. *3*) *Coffee:* scores favorably due to consistent metabolic benefits, but may require adjustments for individuals with sleep disturbance, hypertension, or caffeine sensitivity. The goal is to preserve metabolic intent—lower insulinemic load and inflammation—rather than prescribe specific foods. Individuals avoiding dairy, alcohol, or coffee can adhere to culturally appropriate and mechanistically analogous alternatives.

### Tenet V: incorporate meal context, preparation methods, and food combinations to preserve metabolic integrity

Metabolic responses reflect meals, not isolated foods. In contemporary diets, many foods, particularly preprepared and commercially produced items, are composite and represent multiple food groups. Accordingly, such foods should be evaluated at the level of dishes or meals, accounting for ingredient composition, preparation methods, and food combinations rather than being assigned a single food-group value.

Preparation methods and food combinations can meaningfully alter insulinemic or inflammatory effects: *1*) *Preparation matters—*French fries score negatively due to high-temperature frying, whereas boiled or baked potatoes paired with vegetables may have neutral or favorable metabolic effects. *2*) *Composite dishes can produce emergent metabolic properties:* pizza, for example, may score favorably when dominated by fermented dairy and lycopene-rich cooked tomato sauce. *3*) *Cultural variation in meal structures matters:* curries, stews, noodle soups, tacos, dumplings, and other culturally specific dishes vary widely in composition and preparation. Findings from diverse cohorts [68] show metabolic patterns remain predictive despite this variability. Providing culturally adaptable meal-level guidance helps preserve metabolic integrity while improving feasibility.

### Tenet VI: ensure cultural relevance through community-engaged adaptation

Effective translation requires integrating cultural, social, and environmental contexts: *1*) define food groups broadly but refine examples locally with community input. *2*) Adapt preparation, portioning, and meal patterns collaboratively, ensuring that metabolic directionality, that is, lower insulinemic and inflammatory potential, is maintained. *3*) Use participatory codesign involving clinicians, dietitians, community leaders, patient advocates, and consumers to enhance feasibility, sensory acceptability, and long-term adherence. This approach ensures that metabolic dietary patterns become culturally resonant tools rather than externally imposed prescriptions.

### Tenet VII: incorporate food processing level as an independent layer influencing metabolic effects

Processing level independently modifies insulin and inflammatory pathways. UPFs may elevate insulin response or promote inflammation through additives, emulsifiers, altered matrices, or impacts on the gut microbiome. Conversely, beneficial processing techniques such as fermentation, matrix preservation, or low-temperature cooking can enhance metabolic effects. Food matrix refers to the physical and compositional structure of foods as consumed, including nutrient and non-nutrient organization, processing, and food–food interactions that influence digestion and metabolic response. Implementation should: *1*) identify processing distinctions within food categories (e.g., dairy foods derived from milk but differing in structure and fermentation; whole fruit compared with fruit juice compared with fruit puree – same sugars but very different glycemic/insulinemic responses; whole grains compared with refined grains compared with extruded cereals—same grains but different structure and metabolic effects). *2*) Differentiate beneficial compared with harmful processing, emphasizing fermentation, intact matrices, and minimal additives. *3*) Adapt recommendations to regional food systems, recognizing variability in processing practices, ingredient quality, and supply-chain realities. Considering food processing level as an explicit translational layer improves real-world applicability and strengthens alignment with metabolic pathways.

In conclusion, this translational framework extends current dietary guidance by offering more mechanistically grounded recommendations on food selection, meal composition, preparation methods, and levels of processing to reduce chronic hyperinsulinemia and systemic inflammation, 2 central pathways driving cardiometabolic diseases and several cancers. By integrating metabolic mechanisms with cultural and contextual flexibility, the framework provides a structured pathway for operationalizing low-insulinemic and anti-inflammatory dietary patterns in intervention trials and public health strategies. Although rEDIH and rEDIP were originally derived in United States cohorts, their underlying biological targets are universal, supporting their adaptation to diverse food cultures. Incorporating food processing as an explicit analytic layer further strengthens translational fidelity by capturing the metabolic effects of additives, matrix alterations, and preparation methods that extend beyond nutrient composition. These advances enable tailored interventions for groups likely to benefit most—from individuals with obesity or prediabetes to those transitioning off weight-loss pharmacotherapy or undergoing cancer treatment.

The body of evidence reviewed here affirms the validity of rEDIP and rEDIH as dietary constructs that reflect metabolic dysfunction related to obesity, insulin resistance, and chronic inflammation. Their consistent associations with obesity, type 2 diabetes, CVD, and multiple cancers, together with their moderate correlations with established dietary patterns such as HEI, AHEI, AMED, and DASH, suggest that insulin and inflammatory regulation may represent shared biological pathways through which diverse healthy dietary patterns exert their protective effects. This hypothesis aligns with emerging biomarker and metabolomics data demonstrating that metabolic improvement, and reductions in disease risk, are mediated through coordinated effects on insulin secretion, inflammatory signaling, lipid metabolism, and the gut microbiome, among many other possible mechanisms.

In summary, this perspective provides the first integrative synthesis of metabolic and conventional dietary patterns across nutritional characteristics, biomarker specificity, mechanistic alignment, and predictive ability for chronic disease. We extend prior work by consolidating the body of evidence on metabolic dietary indices, as reflected by rEDIH and rEDIP, articulating the hypothesis that insulin resistance serves as a unifying mechanistic pathway, and outlining a practical translational framework to guide future research, intervention development, and clinical application.

## Author contributions

The authors’ responsibilities were as follows – FKT: created the initial manuscript draft; and all authors: reviewed, edited, and approved the final version.

## Data availability

Data described in the manuscript, codebook, and analytic code will be made available upon request pending application and approval.

## Funding

FKT is funded by American Cancer Society Grants (RSG-20-124-01-CCE, MBGI-23-1155607-01-MBG) and ELG is funded by an American Cancer Society Clinical Research Professor Grant (CPR-23-1014041).

## Conflict of interest

FKT reports financial support was provided by American Cancer Society Inc, which played no role in the conception and design of this perspective. If there are other authors, they declare that they have no known competing financial interests or personal relationships that could have appeared to influence the work reported in this paper.
